# *Dendroctonus rufipennis* (Coleoptera: Curculionidae) responses to 4 doses of 3-methylcyclohex-2-en-1-one (MCH) in baited trapping assays

**DOI:** 10.1093/jee/toaf366

**Published:** 2026-01-20

**Authors:** Jackson P Audley, Christopher J Fettig, Jason E Moan, Jessie Moan, Leif A Mortenson, Agenor Mafra-Neto

**Affiliations:** Department of Plant Sciences, University of California, Davis, CA, USA; Pacific Southwest Research Station, USDA Forest Service, Woodland, CA, USA; Pacific Southwest Research Station, USDA Forest Service, Woodland, CA, USA; Alaska Division of Forestry & Fire Protection, Anchorage, AK, USA; Forest Health Protection, USDA Forest Service, Anchorage, AK, USA; Pacific Southwest Research Station, USDA Forest Service, Placerville, CA, USA; ISCA Inc, Riverside, CA, USA

**Keywords:** antiaggregation pheromone, inhibition, semiochemical repellent, spruce beetle, tree protection

## Abstract

Spruce beetle, *Dendroctonus rufipennis* (Kirby) (Coleoptera: Curculionidae), is the most significant pest of spruce, *Picea* spp. (Pinales: Pinaceae), in western North America. Several doses of 3-methylcyclohex-2-en-1-one (MCH), the primary antiaggregation pheromone of *D. rufipennis*, alone and combined with non-host volatiles have been demonstrated effective for *Picea* protection. Herein, we evaluate the effects of MCH dose on *D. rufipennis* captures in baited trapping assays in Alaska and Colorado, United States. Twenty-five, 12-unit, multiple-funnel traps were baited with a *D. rufipennis* lure (frontalin + MCOL + spruce terpenes; Synergy Semiochemical Corp., Delta, British Columbia, Canada) and randomly assigned to one of 5 treatments in each assay: SBL (baited control); SBL + 1 g MCH; SBL + 3.5 g MCH; SBL + 7 g MCH; and SBL + 10 g MCH. SPLAT MCH (experimental formulation ISR: MCH-001R1, ISCA Inc., Riverside, California, United States), a flowable matrix containing 10.0% MCH by weight, was used in both assays with dose manipulated by the number and size of SPLAT MCH dollops (release points) attached to traps. In both Alaska and Colorado, all MCH doses (1, 3.5, 7, and 10 g) significantly reduced *D. rufipennis* captures compared to SBL. No significant differences were observed among MCH doses. Males and females responded similarly to MCH doses. The implications of these and other results to management of *D. rufipennis* are discussed.

## Introduction

Spruce beetle, *Dendroctonus rufipennis* (Kirby) (Coleoptera: Curculionidae), is the most significant pest of spruce, *Picea* spp. (Pinales: Pinaceae), in western North America (Jenkins et al. 2014, Bleiker and Safranyik 2021). All species and known hybrids of North American spruce are potential hosts; however, most outbreaks occur in large-diameter, mature white spruce, *P. glauca* (Moench) Voss, Lutz spruce, *P.* x *lutzii* Little, and Engelmann spruce, *P. engelmannii* Parry ex. Engelm. (Werner et al. 1977, Holsten and Werner 1990, Ott et al. 2021). Several notable *D*. *rufipennis* outbreaks have occurred in recent years (Fettig et al. 2021). As the climate warms and the frequency and intensity of droughts increase, levels of *Picea* mortality attributed to *D. rufipennis* are projected to increase (Bentz et al. 2010, Hart et al. 2014).

Semiochemical inhibitors can be used to disrupt bark beetle host searching and aggregation behaviors (Seybold et al. 2018). Fettig et al. (2025) reviewed literature on the chemical ecology of *D. rufipennis* with focus on studies investigating semiochemical inhibitors. This literature dates back to the mid-20th century and focuses on *D*. *rufipennis* populations in Alaska, United States, western Canada, and the central and southern Rocky Mountains, United States. Studies demonstrate that the primary antiaggregation pheromone of *D. rufipennis*, 3-methylcyclohex-2-en-1-one (MCH), reduces captures of *D. rufipennis* in baited traps, colonization of felled and standing *Picea*, and mortality of live *Picea* (Fettig et al. 2025). The addition of nonhost volatiles to MCH, including *Acer* kairomone blend (linalool, β-caryophyllene, and (*Z*)-3-hexenol) or PLUS (acetophenone, (*E*)-2-hexen-1-ol, and (*Z*)-2-hexen-1-ol), enhanced these effects in some studies (Hansen et al. 2017, 2019, Audley et al. 2022, 2024, 2025). Several formulations of MCH are registered for *Picea* protection in the United States and Canada (Seybold et al. 2018).

Following the successful formulation of MCH into ISCA’s Specialized Pheromone and Lure Application Technology (SPLAT; Foote et al. 2020), Audley et al. (2022, 2025) reported that 2 doses of SPLAT MCH (experimental formulation ISR: MCH-001R1 containing 3.5 and 7 g of MCH per tree; ISCA Inc., Riverside, California, United States) provided similar levels of *Picea* protection in studies conducted in Colorado, Wyoming, and on the Kenai Peninsula, Alaska, United States. Furthermore, in a comparison of SPLAT MCH and MCH bubble caps on the Kenai Peninsula, 1, 3, and 7 g of MCH provided the same level of *Picea* protection regardless of formulation or dose (Audley et al. 2025). The efficacy of 1 g of MCH reported in Audley et al. (2025) was unexpected and prompted us to investigate the effect of MCH dose on *D*. *rufipennis* captures in baited traps. Understanding dose-dependent responses are important for economic optimization of a semiochemical-based tree protection strategy. We hypothesized that higher doses of MCH (eg, 10 g of MCH per trap) would result in higher levels of inhibition (ie, lower *D*. *rufipennis* captures).

## Materials and Methods

### Gravimetric Release Rate Assay

In our trapping assays, MCH doses were manipulated by the number and size of SPLAT MCH (ISR: MCH-001R1) dollops (release points) attached to traps. To estimate the release of MCH from the 10-g, 17.5-g, and 25-g dollops used in our studies, 6 replicates of each dollop size (*N* = 18) were analyzed gravimetrically (Fettig et al. 2009). Dollops were dispensed into weighing trays and placed in a fume hood. Each dollop was weighed every 3 to 4 d during 17 December 2024 to 7 April 2025. We assume that weight loss is driven by elution of MCH from the SPLAT matrix but recognize there may be additional (inert) volatiles released. Weigh loss data were visually inspected and trimmed to the date at which average weight loss per day was 0.001 to 0.002 mg. A linear model was fitted to the trimmed data for each dollop size. Analyses were conducted using the Stats package with R Statistical software (version 3.6.3) via RStudio (Version 1.2.5033, R Core Team 2020).

### Trapping Assays

Two trapping assays were conducted in 2025, one near Denali National Park, Alaska (elevation 644.7 to 648.3 m, centered at 63.620°N, 148.772°W) and another below Cottonwood Pass near Buena Vista, Colorado (elevation 3519.8 to 3555.2 m, centered at 38.812°N, 106.403°W). In each assay, a 25-trap array consisting of 3 or 4 transects was constructed with adjacent trap locations spaced ≥30 m. At each location, a 1-m long rebar was hammered into the ground. A 2.4-m long steel conduit pole was placed over the rebar from which a 12-unit, multiple-funnel trap was hung placing the bottom of the trap ∼95 cm above the forest floor. One *D. rufipennis* lure (SBL; Product #3123, Synergy Semiochemical Corp., Delta, British Columbia, Canada) consisting of frontalin (1.25 mg/d at 20 °C), MCOL (3 to 5 mg/d at 25 °C), and spruce terpenes was attached to each trap. Traps were then randomly assigned to one of 5 treatments (*n *= 5): SBL (baited control); SBL + 1 g MCH; SBL + 3.5 g MCH; SBL + 7 g MCH; and SBL + 10 g MCH (Table 1). All semiochemicals were attached to each trap between the sixth and seventh funnels. SPLAT MCH was used in both assays with dollops dispensed into 9-cm by 9-cm plastic weighing trays which were then attached to the top edge of a trap funnel with a binder clip. Insecticide strips (Hot Shot No Pest Strips, 18.6% 2,2-dichlorovinyl dimethyl phosphate, Spectrum Brands Inc., Middleton, Wisconsin, United States) were placed in each collection cup to kill captured insects. Trap placement was randomly assigned. Traps were left in place for ∼24 h after which captures were collected and trap locations were rerandomized. Trapping was conducted 16 to 26 June 2025 in Alaska and 7 to 15 July 2025 in Colorado.

**Table 1.** Mean gravimetric release (mg/d ± SEM) of 3-methylcyclohex-2-en-1-one (MCH) from 10-g, 17.5-g, and 25-g dollops of SPLAT MCH^a^ under laboratory conditions at 20 °C

Dollop size	**Weight loss (mg/d)**	**Longevity (d)^b^**	Treatment	Number, size of dollops	Total release (mg/d)^c^
**10 g**	28.0 ± 3.5	28	1 g MCH	1, 10-g dollop	28 mg/d
**17.5 g**	27.6 ± 2.1	51	3.5 g MCH	2, 17.5-g dollops	55.2 mg/d
**25 g**	30.1 ± 1.9	69	7 g MCH	4, 17.5-g dollops	110.4 mg/d
			10 g MCH	4, 25-g dollops	120.4 mg/d

a SPLAT MCH (ISR: MCH-001R1, ISCA Inc., Riverside, California, U.S.) contains 10.0% MCH by weight. For example, a 10-g dollop of SPLAT MCH contains 1 g of MCH.

b Longevity of release cropped when average weight loss was 0.001 to 0.002 mg/d.

c Mean values were used to calculate the total release of MCH per treatment.

To describe general stand conditions, 0.041-ha circular plots were established at each odd-numbered trap location (*n* = 13 per assay) with the conduit pole serving as the center of the plot. All live trees ≥10.2 cm diameter at breast height (dbh, 1.37 m in height) in Alaska and ≥12.7 cm dbh in Colorado were identified to species and the dbh was recorded. Numbers of *Picea* colonized by *D*. *rufipennis* in 2024 and 2025 were identified based on the presence of pitch tubes and/or boring dust. Numbers of *Picea* killed by *D*. *rufipennis* since 2023 were identified based on the presence of crown fade and needle loss on *Picea* that had evidence of colonization by *D*. *rufipennis*.

Three days (days 2, 7, and 9) were excluded from analyses in Alaska (*n* = 7 collections) and one day (day 8) in Colorado (*n* = 7 collections) as cool temperatures (<14.5 °C; Holsten and Hard 2001), wind, and/or rain contributed to low (<10 total *D. rufipennis*) trap captures. All *D. rufipennis* were sexed based on examination of the seventh abdominal tergite (Lyon 1958). Captures of total *D. rufipennis*, male *D. rufipennis*, and female *D. rufipennis* were compared by fitting generalized linear models with a negative binomial distribution and a log link (lme4 package). Treatment, day, trap location, and their interactions were considered in each model. Model selections were informed by Akaike information criterion values and likelihood ratio tests (Zuur et al. 2009). Multiple means comparisons were made with post hoc pairwise, least squares means (emmeans package) tests with the Tukey correction (α  =  0.05). All analyses were conducted using R Statistical software (version 3.6.3) via RStudio (Version 1.2.5033, R Core Team 2020).

## Results

### Gravimetric Release Rate Assay

Mean air temperature in the fume hood during the release rate assay was 20.2 ± 0.1 °C (mean ± SEM reported in all cases). Per day weight loss data were trimmed at 28 d for 10-g dollops, 51 d for 17.5-g dollops, and 69 d for 25-g dollops. Linear models fit each dataset well with adjusted *R*^2^ values of 0.95, 0.64, and 0.67 for the 10-g, 17.5-g, and 25-g dollops, respectively. Release rates varied little among dollop sizes (Table 1). In fact, pairwise, Welch’s t-tests revealed no differences in the slopes of the fitted linear models (all *t* ≤ 0.17, df > 81.9, *P *≥ 0.86). Larger dollops increased the longevity of MCH release. The 17.5-g dollops released MCH ∼82% longer than the 10-g dollops and the 25-g dollops released MCH ∼146% longer than the 10-g dollops (Table 1).

### Trapping Assay—Alaska

The trapping assay in Alaska was conducted in a *P*. *glauca* forest with 1,083.5 ± 188.3 *P*. *glauca* per hectare and 18.9 ± 2.7 m^2^ of basal area per hectare. No other tree species were represented. Mean dbh was 15.6 ± 0.7 cm. On average, 42.9 ± 5.2% of *P*. *glauca* were colonized by *D*. *rufipennis* in 2024 and 2025, and 16.5 ± 2.3% were killed since 2023. A total of 239 *D. rufipennis* (89 males, 150 females) were captured over 7 d.

**Fig. 1.**
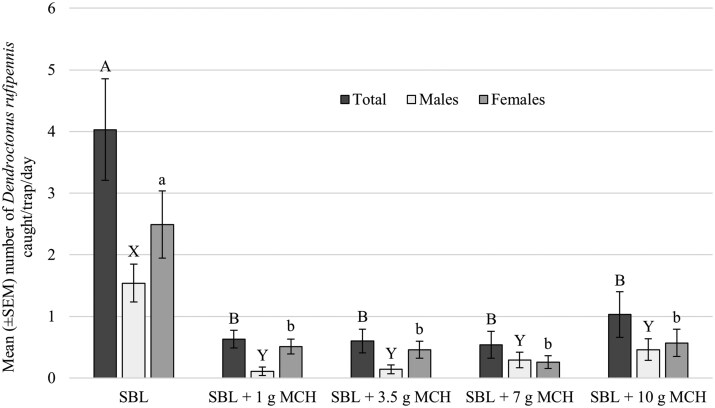
Mean (± SEM) numbers of total *Dendroctonus rufipennis*, male *D*. *rufipennis*, and female *D*. *rufipennis* captured per trap per day in 12-unit, multiple-funnel traps baited with an enhanced trapping lure (SBL = frontalin + MCOL + spruce terpenes; Product #3123, Synergy Semiochemical Corp., Delta, BC, Canada). A formulation of ISCA’s Specialized Pheromone and Lure Application Technology (SPLAT) MCH (ISR: MCH-001R1, ISCA Inc., Riverside, California, United States), a flowable matrix containing 10.0% 3-methylcyclohex-2-en-1-one (MCH) by weight, was used with dose manipulated by the number and size of SPLAT MCH dollops (release points) attached to traps. Traps were deployed near Denali National Park, Alaska, United States, 16 to 26 June 2025. Different letters among bars of the same color indicate significantly different means (α  =  0.05) based on negative binomial generalized linear modeling and least squares means comparisons with a Tukey correction.

The final model selected for total captures of *D. rufipennis* retained treatment, day, and trap location without any interactions between fixed effects. Day (z = −4.04, *P *< 0.01), SBL treatment (z = 4.31, *P *< 0.01), and intercept (z = 2.65, *P *= 0.01) were all significant in the model. Models for male *D. rufipennis* and female *D. rufipennis* only retained treatment and day. For males, day (z = −3.42, *P *< 0.01), SBL treatment (z = 3.42, *P *< 0.01), MCH 3.5 g treatment (z = -1.99, *P *= 0.05), and MCH 1 g treatment (z = −2.19, *P *= 0.03) were significant. For females, day (z = −3.3, *P *< 0.01) and SBL treatment (z = 4.57, *P *< 0.01) were significant. Mean numbers of *D. rufipennis* caught per trap per day were significantly different for total (χ^2^ = 37.6, df = 4, *P *< 0.01), males (χ^2^ = 42.3, df = 4, *P *< 0.01), and females (χ^2^ = 33.9, df = 4, *P *< 0.01). Fewer total *D. rufipennis*, male *D. rufipennis*, and female *D. rufipennis* were captured in the MCH treatments compared to SBL (Fig. 1). No differences were observed among MCH doses.

### Trapping Assay—Colorado

The trapping assay in Colorado was conducted in a *P*. *engelmannii-*subalpine fir, *Abies lasiocarpa* (Hook.) Nutt. (Pinales: Pinaceae), forest with 790.7 ± 52.3 trees per hectare and 54.9 ± 3.8 m^2^ of basal area per hectare. About 82.5% of trees were *P*. *engelmannii*. Mean dbh of *P*. *engelmannii* was 31.2 ± 0.8 cm. On average, 35.9 ± 5.9% of *Picea* were colonized by *D*. *rufipennis* in 2024 and 2025, and 15.0 ± 3.3% were killed since 2023. A total of 701 *D. rufipennis* (299 males, 402 females) were captured over 7 d.

**Fig. 2.**
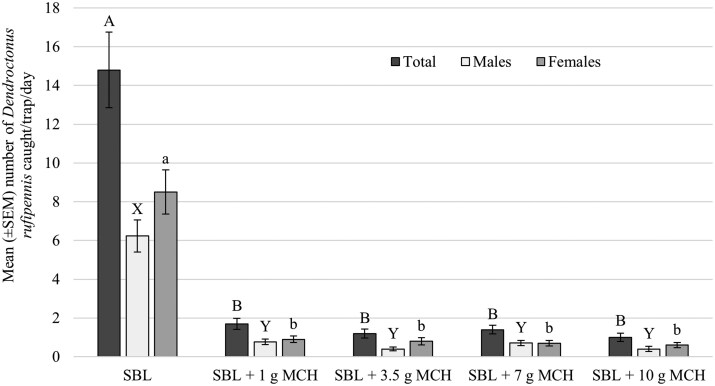
Mean (± SEM) numbers of total *Dendroctonus rufipennis*, male *D*. *rufipennis*, and female *D*. *rufipennis* captured per trap per day in 12-unit, multiple-funnel traps baited with an enhanced trapping lure (SBL = frontalin + MCOL + spruce terpenes; Product #3123, Synergy Semiochemical Corp., Delta, BC, Canada). A formulation of ISCA’s Specialized Pheromone and Lure Application Technology (SPLAT) MCH (ISR: MCH-001R1, ISCA Inc., Riverside, California, United States), a flowable matrix containing 10.0% 3-methylcyclohex-2-en-1-one (MCH) by weight, was used with dose manipulated by the number and size of SPLAT MCH dollops (release points) attached to traps. Traps were deployed near Cottonwood Pass, Colorado, United States, 7 to 15 July 2025. Different letters among bars of the same color indicate significantly different means (α  =  0.05) based on negative binomial generalized linear modeling and least squares means comparisons with a Tukey correction.

The final model selected for total captures of *D. rufipennis*, male *D. rufipennis*, and female *D. rufipennis* each retained treatment and day without interactions between fixed effects. In each case, SBL treatment and day were significant: total SBL treatment (z = 11.44, *P *< 0.01), day (z = −3.19, *P *< 0.01); male SBL treatment (z = 8.86, *P *< 0.01), day (z = −2.02, *P *= 0.04); female SBL treatment (z = 9.81, *P *< 0.01), day (z = −3.08, *P *< 0.01). Mean trap captures were different for total *D. rufipennis* (χ^2^ = 79.9, df = 4, *P *< 0.01), male *D. rufipennis* (χ^2^ = 77.88, df = 4, *P *< 0.01), and female *D. rufipennis* (χ^2^ = 77.36, df = 4, *P *< 0.01). Fewer total *D. rufipennis*, male *D. rufipennis*, and female *D. rufipennis* were captured in the MCH treatments compared to SBL (Fig. 2). No differences were observed among MCH doses.

## Discussion

We were surprised to find no differences in captures among MCH doses evaluated in our studies (Figs 1 and 2). However, our findings support several studies that found different doses of MCH provided similar levels of *Picea* protection. For example, Hansen et al. (2017) compared infestation rates of *P*. *engelmannii* on 0.64-ha plots in New Mexico and Utah treated with 20, 40, and 80 g of MCH per ha. The probability of more severe *D*. *rufipennis* attacks was reduced by MCH by ∼50% compared to the control (control = 0 g MCH). No differences were observed among MCH doses. Audley et al. (2022, 2025) reported that doses of MCH from 1 to 7 g per study tree provided similar levels of protection for *P*. x *lutzii* in Alaska and also for *P*. *engelmannii* in Colorado and Wyoming. MCH is also the primary antiaggregation pheromone of Douglas-fir beetle, *Dendroctonus pseudotsugae* Hopkins, and is commonly used to protect Douglas-fir, *Pseudotsuga menziesii* (Mirb.) Franco (Pinales: Pinaceae), from mortality attributed to *D*. *pseudotsugae*. Ross et al. (1996) applied 20, 40, and 60 g of MCH per hectare and reported the percentage of *Ps*. *menziesii* ≥20 cm dbh that were mass attacked by *D*. *pseudotsugae* was lower on MCH-treated plots than on the control plots (control = 0 g MCH). No differences were observed among MCH doses. Captures of *D*. *pseudotsugae* at the center of MCH-treated plots were lower than at the center of control plots, but there were no differences in captures among MCH doses (Ross et al. 1996).

There are examples of dose-dependent responses of bark beetles to antiaggregation pheromones and other inhibitors. For example, Miller et al. (1995) demonstrated that the effect of verbenone on *D*. *ponderosae* and two *Ips* spp. (Coleoptera: Curculionidae) was dose dependent, with the highest dose having the strongest inhibitory effect. Gomez et al. (unpublished data; JPA, CJF, LAM, and AMN coauthors) found a higher dose of verbenone (3.5 vs. 7 g per trap) increased inhibition of southwestern pine beetle, *D*. *barberi* Hopkins, when combined with PLUS (acetophenone, (*E*)-2-hexen-1-ol, and (*Z*)-2-hexen-1-ol). Verbenone is the primary antiaggregation pheromone of mountain pine beetle, *D*. *ponderosae* Hopkins, western pine beetle, *D*. *brevicomis* LeConte, and southern pine beetle, *D*. *frontalis* Zimmermann) and inhibits the response of these and several other bark beetle species to baited traps and trees (Seybold et al. 2018, Gaylord et al. 2023, Frühbrodt et al. 2024).

Byers (2013) demonstrated that antennal receptor responses to bark beetle pheromones are best described by kinetic functions which often yield a sigmoidal response curve on a logarithmic scale (eg, *see* Fig. 5 in Byers 2013). Differences in responses are most often observed between release rates with at least an order of magnitude difference. In our study, doses of ≥1 g MCH per trap may be above the threshold where even an order of magnitude increase in dose no longer yields a change in response (ie, the middle section of a sigmoid curve in Fig. 5 in Byers 2013). However, it is important to note that while our minimum and maximum doses varied by 10X (from 1 to 10 g of MCH per trap), release rates varied by only 4.3X (Table 1). Unlike MCH, verbenone is also produced within hosts by auto-oxidation of α-pinene, which may signal a decline in host tissue (phloem) quality to some *Dendroctonus* spp. (Frühbrodt et al. 2024). The lack of source specificity for verbenone may help explain the dose-dependent responses observed in some verbenone studies.

Although not evaluated, differences in inhibition among MCH doses (and release rates) could also result from differences in the longevity of MCH release among doses in assays conducted over longer periods of time. To that end, compared to 10-g dollops of SPLAT MCH, 17.5-g and 25-g dollops increased the length of MCH release by 23 and 41 d (in the laboratory at 20 °C), respectively. The movement of MCH within the SPLAT matrix occurs by diffusion and follows Fick’s First Law (Mafra-Neto et al. 2013), which states that molecules move down their concentration gradients at a rate that is directly proportional to their concentration gradient. This rate is influenced by temperature, with higher rates occurring during higher temperatures. Mean daily temperatures within the range of *D*. *rufipennis* during the flight period are often below 20 °C (eg the mean daily temperature for June near our study site in Alaska is 12.2 °C) extending the longevity of MCH release under field conditions compared to data reported from our release rate assay. As such, it may be worthwhile investigating the release of MCH from SPLAT (and other devices) at lower temperatures (eg 14 to 16 °C). Our results support the SPLAT MCH doses (3.5 and 7 g, applied as 2 or 4 17.5-g dollops per tree) evaluated by Audley et al. (2022, 2025) for *Picea* protection as *D. rufipennis* dispersal and host searching typically begins between mid-May and mid-June and extends to late-summer, temperatures permitting (Jenkins et al. 2014, Bleiker and Safranyik 2021). The 10-g dollops may not release MCH long enough to impart *Picea* protection for the full flight period on warmer sites (eg on southern slope aspects at lower elevations). Finally, it should be noted that we observed no differences between male and female responses to MCH doses. Female *D. rufipennis* initiate host colonization and are later joined by males (Jenkins et al. 2014, Bleiker and Safranyik 2021). As such, inhibition of females is of utmost importance for *Picea* protection.

The next step is to evaluate the efficacy of SPLAT MCH for protecting *Picea* at the stand-level. The results reported herein and in Audley et al. (2022, 2024, 2025) will help inform these efforts and future research. For example, Audley et al. (2025) reported the range of inhibition provided by 35 g of SPLAT MCH (two 17.5-g dollops of SPLAT MCH) for *D. rufipennis* was 12 m in Colorado and 4 m in Alaska. These data will be used to inform the spacing of MCH point sources in stand-level studies while recognizing that additional studies evaluating the effects of MCH doses and SPLAT MCH dollop sizes on the range of inhibition of *D. rufipennis* are warranted.
